# The Impact of Pollution Fee Reform on the Emission of Water Pollutants: Evidence from Manufacturing Enterprises in China

**DOI:** 10.3390/ijerph191710660

**Published:** 2022-08-26

**Authors:** Zhe Yang, Zhenwu Xiong, Wenhao Xue, Yuhong Zhou

**Affiliations:** School of Economics, Qingdao University, Qingdao 266071, China

**Keywords:** water pollution, pollution fees, environmental protection tax, manufacturing enterprise, multiperiod DID

## Abstract

With the development of China’s industrial economy and urbanization, water pollution has become serious and gradually exposed to the public. The pollution fee policy is an important tool to force enterprises to reduce pollution. This study used the panel data of manufacturing enterprises during 2006–2013 and the multiperiod difference in differences (DID) method to systematically analyze the impact of water pollution fee reform on emissions of manufacturing enterprises in China. In general, enterprises facing improved pollution fee collection standards reduce COD emissions by approximately 4.1%. However, significant location heterogeneities are captured in China. The rising water pollution fees have promoted the emission reduction of enterprises in northern China and resource-based cities, but the effect is not significant in southern China and nonresource-based cities. Furthermore, the mechanism analysis shows that enterprises mainly reduced emissions through terminal treatment and reducing production. This study provided micro evidence for research on the effect of pollution fee reform and supplied a reference for the improvement of the environmental protection tax system in China.

## 1. Introduction

Water, as the source of life, is an important condition for human survival and development. The rapid expansion of human activities has brought about profound changes in the natural environment on which we live. It also reminds us that while pursuing economic development, we must also consider the protection of the natural environment [1]. Over the past 40 years of reform and opening up, the economy has maintained rapid growth in China. However, high energy consumption and high pollution development have also caused serious environmental pollution problems [2,3,4]. The rapid development of urbanization and industrialization in the Karst region of Southwest China has caused regional water shortage and water pollution [5]. China is currently in the modernization stage of industrialization. Industrial enterprises are the core carriers of creating social and economic wealth, and they are also the takers of natural resources [6]. Industrial wastewater has become the main source of water pollution [7,8]. Water pollution not only restricts industrial and agricultural production but also has an impact on people’s living environment and physical health. In the Karst region of Central China, pollutants can flow into groundwater, causing contamination of underground systems [9,10] and potential risks to human health [11]. Water pollution mainly can be divided into black and odorous water caused by ammonia nitrogen (NH_3_−N), biochemical oxygen demand (BOD_5_), and chemical oxygen demand (COD) [12]; high concentrations of eutrophication pollution caused by nitrogen and phosphorus substances [13]; and toxic water pollution mainly caused by heavy metals, etc. [14]. Therefore, the problem of water pollution has aroused the attention of the Chinese government. To control water pollution and encourage enterprises to reduce industrial sewage emissions, China has promulgated a series of environmental regulations, and the types of policies have gradually diversified [15]. Among them, market-based environmental regulation means that the government encourages enterprises to consciously carry out energy-saving and emission-reduction behaviors to protect the environment through market-oriented measures such as pollution discharge fees and environmental protection taxes [16]. As a market-oriented environmental measure, the pollution fee reform aims to improve water quality by internalizing the external costs and incentivizing enterprises to reduce water-pollutant emissions.

In terms of the impact of pollution fees on pollutant emissions, many scholars had extensive discussions, and there are two views on this issue: one is that pollution fees are not conducive to pollution reduction. Lin found that China’s pollution fee collection system is not obvious to reduce the COD of factories [17]. Andrew et al. studied the impact of the double dividend (i.e., economic dividend and green dividend) of Nordic economies from the perspective of the energy, pollution, and resource tax and found that only the energy tax showed the double dividend [18]. Alfred constructed a simple profit function model to analyze the causation between effluent charges and environmental damage and then found that there was no necessary negative causation between effluent charges and environmental damage [19]. Hu et al. estimated the marginal cost of air- and water- pollutant emissions from key industries in Yunnan Province by setting high, medium, and low tax brackets; they pointed out that the environmental protection tax rates in China are generally low, and suggested that the government needs to appropriately increase the environmental tax rate so that the charging standard is close to the external cost of the enterprise [20].

However, compared with the above view, the hypothesis that pollution fees can promote pollution reduction is more agreed upon by scholars. Berbel et al. explored the implementation of market-based environmental regulations (such as water taxes and trading) in European countries, and they found that water taxes can reduce water pollution [21]. Dasgupta also found that the imposition of pollution fees can reduce the level of COD and suspended solids [22]. Allen, through a study of Colombia’s effluent charge system, found that in the first 5 years, the implementation of the program was limited in many areas, but in some areas, it played a role in reducing pollution [23]. Buhari et al. found that environmental taxes can effectively reduce CO_2_ emissions in G7 countries [24]. Wang and Wheeler studied the effects of China’s pollution fee and found that charging pollution fees for air and water pollution can significantly promote enterprises to reduce pollutant emissions [25]. Dong et al. found that the levy of pollution fees contributes to COD emissions, showing a significant inverted U-shaped relationship, and can further reduce COD emissions by promoting technological innovation [26]. He and Zhang used the DID method to study the pollution control effect of the environmental tax pilot policy in the Taihu Lake Basin and found that the total COD emissions of enterprises located in the Taihu Lake Basin decreased by about 7% compared with the data from 2007 [27].

Compared with the previous literature, the marginal contribution of this paper lies in the following aspects. (1) Most researchers have discussed the environmental dividends of environmental regulation at the macro level. This study took manufacturing enterprises as the research sample to systematically evaluate the policy effect of the reform of pollution fee standards and provide micro-enterprise-level evidence for the environmental tax policy. (2) The previous literature mostly focused on the impact of the total amount of pollution fees on the discharge of three kinds of industrial wastes [26,28,29] or the impact of SO2 pollution fee reform on exhaust emissions [30]. This study took water pollutants as the research object and explored the impact of water pollution fee reform on the emission of water pollutants. (3) Few studies have explored the specific measures of polluting enterprises in response to pollution fee reform. This study further decomposed the emission reduction behavior of enterprises into process control, end-of-pipe control and production reduction, and explored the influencing mechanism of pollution fee reform, providing a reference for improving environmental protection tax policy.

## 2. Policy Background and Mechanism Analysis

### 2.1. Policy Background

Pollution fees originated from the Pigouvian tax theory [31]. That is, the government can internalize the negative externality costs of enterprises by levying taxes on polluting enterprises, thereby stimulating the environmental responsibility awareness of enterprises and motivating and guiding enterprises’ environmental protection behavior.

**Figure 1:**
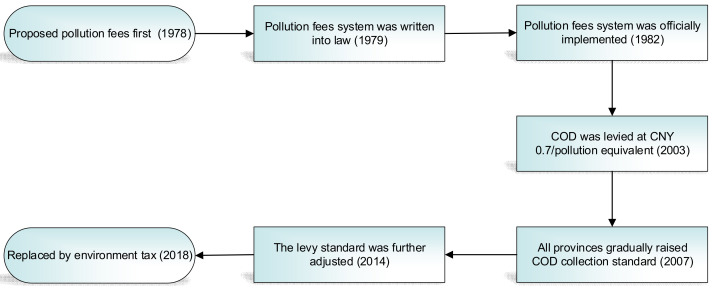
Development history of the pollution fee system in China.

As seen in Figure 1, at the end of 1978, the Chinese government proposed to “charge polluters for discharging pollutants” for the first time. In September 1979, the pollution fee system was legally established. In July 1982, the Chinese government officially implemented the Interim Measures for the Collection of Pollution Fees, marking the official start of China’s nationwide implementation of the pollution fee system. After the promulgation of Regulations on the Administration of Collection and Use of Pollutant Discharge Fees in 2003, the pollution fee system underwent significant changes. The pollution fees have changed from charging by the concentration to charging by the total amount. The collection standard is unified across the country, and a normalized measurement method for pollutants has been established. The fee standard is CNY 0.7 per water pollution equivalent. In 2007, the Chinese government promulgated the Notice of Printing and Distributing Comprehensive Work Plans for Energy Conservation and Emission Reduction, requiring all localities to raise the standards of COD pollutant emission fees according to actual conditions. In 2014, the standard of pollution fees was further reformed. For COD pollutants, the standard was raised from CNY 0.7 to CNY 1.4 per pollution equivalent.

After the implementation of China’s Environmental Tax Law on 1 January 2018, the pollution fee system, which has been levied for nearly 40 years, was officially replaced by the environmental protection tax. Although it was implemented at a relatively low standard [32], it also played a pivotal role in China’s environmental protection for a certain period, such as increasing the public attention toward water pollution and raising special funds for environmental protection [33].

### 2.2. Mechanism Analyses and Research Hypotheses

By internalizing the negative externality cost of pollution emissions, environmental regulation forces enterprises to actively seek a green development path and reduce their pollutant emissions, thereby improving the environment [34,35]. Accordingly, this paper proposed Hypothesis 1.

**Hypothesis** **1:** *Water pollution fee reform encourages enterprises to reduce their water-pollutant emissions*.

The behavior choices of enterprises in response to environmental regulation may be different. When enterprises face environmental regulations, in addition to reducing production, they also use technologies, which are usually subdivided into process and end-of-pipe control [36]. Process control uses cleaner raw materials, improved product designs, and production technology innovations from the source to reduce emissions. End-of-pipe control curbs pollution emissions by introducing pollution treatment facilities [37]. To deeply examine the behavior choices of enterprises in response to environmental regulation, the methods of Ren et al. and Qian et al. were used in this study [38,39]. In Equation (1), the COD emission was decomposed into three parts.

(1) $$\ln(\mathrm{COD}\;\mathrm{emission})=\ln(Y)+\ln(\frac{\mathrm{COD}\;\mathrm{generation}}{Y})+\ln(\frac{\mathrm{COD}\;\mathrm{emission}}{\mathrm{COD}\;\mathrm{generation}})$$

Among them, COD emission represents the emission of COD, COD generation represents the amount of COD produced, and Y represents the gross industrial output value. COD generation/Y represents the process control of pollutants in the enterprise. The reason is that enterprises can reduce the amount of pollutants produced per unit of industrial output value by adopting advanced technology and equipment, improving management, recycling, and other process measures. The smaller the index, the better the process control. COD emission/COD generation represents the end-of-pipe control. The reason is that enterprises use centralized sewage treatment facilities for end-to-end control, which can reduce the amount of pollutants discharged under the condition that the amount of pollutants produced remains unchanged. The smaller the index, the better the end-of-pipe control.

**Figure 2:**
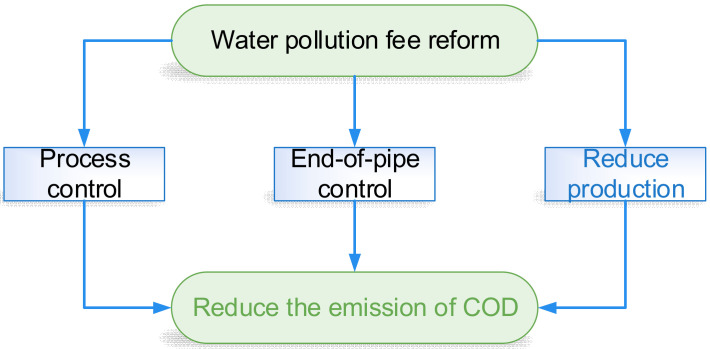
Approach to enterprise emission reduction.

Through mechanism analysis, it is found that enterprises have three choices when facing environmental regulation (i.e., reduce production, process control, and end-of-pipe control), as shown in Figure 2. Among these three approaches, reducing production is a direct way to reduce pollution emissions. Process control requires large-scale adjustments in the production process, while end-of-pipe control costs are relatively low, and the effect of emission reduction is obvious. Based on this, this paper put forward Hypothesis 2.

**Hypothesis** **2:** *Water pollution fee reform encourages enterprises to carry out end-of-pipe control and reduce production, but the impact on process control is not significant*.

## 3. Models and Data Collection

### 3.1. Basic Model

The purpose of this study was to explore the impact of the adjustment of the pollution fee standard on water pollution emissions. We select COD emissions as the core explained variable. In order to control the impact of some observable factors on COD emissions, we also incorporated the gross industrial output, industrial water consumption, industrial wastewater emissions, industrial wastewater treatment, and NH3−N emissions. To effectively avoid the interference of heteroscedasticity, each variable was processed with a natural logarithm.

A multiperiod difference in differences (DID) model was designed for empirical testing in this study as follows. Based on this model, the policy effects and cumulative effects of policies over time can be more accurately analyzed [40].

(2) $$LnCOD_{it}=\alpha_{0}+\alpha_{1}COD\_DID+\beta X_{it}+\delta_{t}+\vartheta_{i}+\gamma_{ht}+\varepsilon_{it}$$

where *LnCOD* refers to the explained variable (i.e., COD emissions). *COD_DID* refers to the core explanatory variable, indicating whether enterprise i is subject to the adjustment of the pollution fee standard in year t. $X_{it}$ represents a series of control variables. $\delta_{t}$ represents the time fixed effect, $\vartheta_{i}$ represents the individual fixed effect, $\gamma_{ht}$ represents the industrial-time fixed effect, and $\varepsilon_{it}$ is a random disturbance term. $\alpha_{1}$ reflects the difference between the COD emission changes in the enterprise in the area that is affected by the reform of the pollution fee standard and the enterprise in the area not affected by the pollution fee standard reform. If $\alpha_{1}$ is significantly negative, it represents the fact that the reform of the pollution fee standard is beneficial to the reduction of COD by enterprises. *i*, *h*, and *t* represent the enterprise, industry, and time, respectively. In addition, this paper chose to cluster standard errors to the four-digit industry level.

### 3.2. Variable Selection and Data

The research sample data used in this study came from China’s manufacturing enterprises for the period 2006–2013. The samples of Tibet, Hong Kong, Macao, and Taiwan were removed because of missing relevant data used in this study. Finally, the panel data of 326,368 samples from 2006 to 2013 were obtained, and Table 1 shows the descriptive statistics of the relevant variables used in this study.

**Figure 3:**
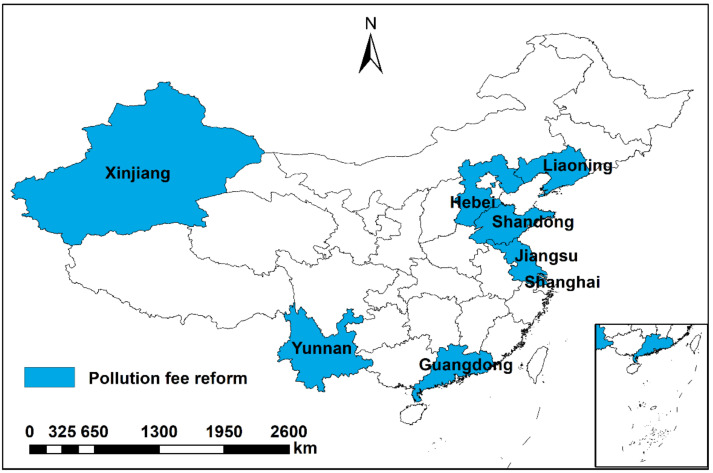
Spatial distribution of the pollution fee reform areas in China during 2006–2013.

During the sample period, the areas where pollution fee reform occurred included Jiangsu, Shanghai, Hebei, Shandong, Yunnan, Guangdong, Liaoning and Xinjiang provinces. It can be seen in Figure 3. In 2006, the GDP, number of industrial enterprises, and COD emissions of these eight provinces accounted for 46.9%, 49.6% and 34.2% of the national total, respectively. Therefore, the study area is representative.

#### 3.2.1. Explained Variable

As COD is the main pollutant in the water body, can lead to water eutrophication, and is toxic to fish and some aquatic organisms, COD emissions were selected as the explained variable in this paper. Enterprises with at least three consecutive years of data and at least 1 year of data before and after the implementation of the policy are retained in this study. The emission data of the manufacturing enterprises are available in the enterprise green development database of China’s microeconomic data query system. For the sake of robustness and controlling the influence of extreme values, this study also performed winsorization on the 1% and 99% quantiles of all continuous variables in the regression test (the same was carried out for the other variables).

#### 3.2.2. Core Explanatory Variables

The adjustment of the COD pollution fee standard (denoted by COD_DID) is a dummy variable and represents the policy shock of the pollution fee reform. The value of the variable COD_DID is 1 in the year that the pollution fee standard reform occurred in the province where the enterprise is located and in the following years; otherwise, the value is 0. The data of the reform of the COD pollution fee standard were manually collected through the official websites of each government. The provinces whose COD pollution fee collection standards have been adjusted during the sample period are shown in Table 2.

#### 3.2.3. Control Variables

Some variables also need to be controlled due to the influence on pollution emissions of enterprises and policy implementation. Here, we collected five control variables from the data available in the enterprise green development database of China’s microeconomic data query system. (1) The gross industrial output value (denoted by LnGiov). The gross industrial output value of an enterprise has a direct impact on emissions. The larger the gross industrial output value becomes, the more likely it is to emit more water pollutants [15]. Drawing on the method of Ren et al. [39], we deleted the observations with a gross industrial output value of less than CNY 10,000. (2) Industrial water consumption (denoted by LnIwc): in general, industrial pollutant emissions and industrial water consumption have a positive relationship. (3) Industrial wastewater treatment volume (denoted by LnIwtv): the more the industrial wastewater that is treated, the less the industrial pollutant emissions there should be. (4) Industrial wastewater emissions volume (denoted by LnIwev): since industrial water pollutants are contained in industrial wastewater, the more the industrial wastewater that is discharged, the larger the industrial pollutant emissions there should be. (5) NH3−N emissions (denoted by LnNH3-N_e): there is a certain relationship between COD and NH3−N emissions [30].

## 4. Results and Analysis

### 4.1. Benchmark Regression

#### 4.1.1. Parallel Trend Test

Before conducting the multiperiod DID, a parallel trend test must be performed to avoid biased results as much as possible. Therefore, this paper used dynamic effect detection to ensure that the COD emissions of the enterprises in the experimental group and the enterprises in the control group have the same trend before the implementation of the policy. Referring to the practice of Liu [40], the specific regression equation is as follows:

(3) $$LnCOD_{it}=\beta_{0}+\beta_{1}pre_{it}^{-3}+\beta_{2}pre_{it}^{-2}+\beta_{3}current_{it}+\beta_{4}post_{it}^{+1}+\ldots \ldots \beta_{8}post_{it}^{+5}+\delta_{t}+\theta_{i}+\gamma_{ht}+\varepsilon_{it}$$

**Figure 4:**
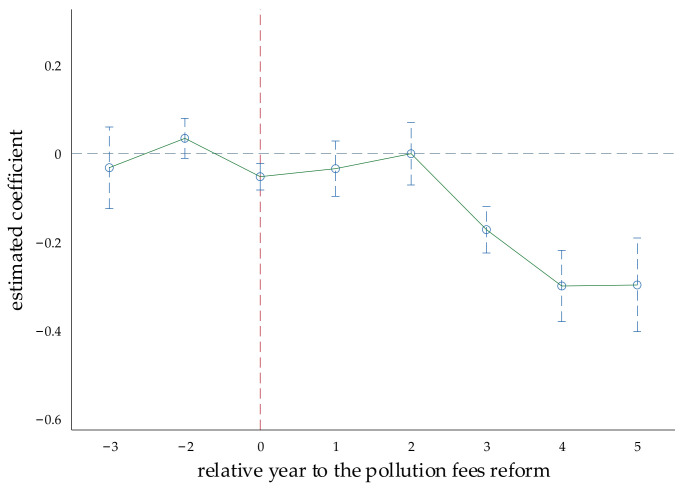
Results of the parallel trend test.

Among them, $pre_{it}^{+j}$(j = −3, −2), $current_{it}$, and $post_{it}^{+k}$(k = +1, …, +5) represent the fact that in the j, k, and current periods of policy implementation, respectively, the index in the experimental group enterprises is 1; otherwise, it is 0. j represents the year before the policy is implemented. For example, $pre_{it}^{-3}$ represents the third year before the policy implementation of the experimental group enterprises. In this year, the indicator is 1, and in the other times, the indicator is 0. The control group enterprises have an indicator of 0 at all times. In this paper, the year before the policy occurred was taken as the base year; then, the regression coefficient of $post_{it}^{+k}$ represented the impact of the kth period of policy implementation on the enterprises’ COD emissions relative to the year before the implementation of policy. The dynamic trend can be seen in Figure 4.

Figure 4 illustrates that the trends of COD emissions between the experimental and control groups have no obvious difference before the policy implementation, which conforms to the parallel trend. Moreover, we also found that 2 years after the implementation of the policy, there is a significant downward trend. The possible reason is that the policy has a certain time lag, and it also takes time for enterprises to adjust their production behavior.

#### 4.1.2. Benchmark Regression Results

The benchmark regression results of this study are shown in Table 3. Column (a) shows the regression results without control variables. Without control variables, the policy (i.e., COD_DID) can significantly reduce COD emissions (i.e., LnCOD). Column (b) shows the regression results after adding a series of control variables. The policy still has a significant negative impact on COD emissions, and the coefficient of policy shock variable is −0.041, which means that compared with enterprises with unchanged COD pollution fee collection standards, enterprises with improved collection standards reduce COD emissions by an average of 4.1%, and it is significantly established at the 5% level. Therefore, Hypothesis 1 is supported. The increase in the pollution fee standard can force enterprises to reduce their COD emissions, which is beneficial to improving the water environment.

For control variables, the gross industrial output value (i.e., LnGiov), industrial water consumption (i.e., LnIwc), and industrial wastewater emissions (i.e., LnIwe) have a significant positive impact on COD emissions. However, the industrial wastewater treatment volume (i.e., LnIwtv) has a negative impact on COD emissions. The results are in line with the expectations. In addition, there is a positive relationship between COD emissions and NH3−N emissions (i.e., NH3-N_e). The reason may be that NH3−N and COD are both pollutants of industrial wastewater.

### 4.2. Robustness Test

#### 4.2.1. PSM-DID

To further ensure the accuracy of the results, this paper used the propensity score matching multiperiod DID (PSM-DID) method for testing. The reason is that the treatment group in this paper was the enterprises whose pollution fee standard was adjusted during the sample period, and the control group was the enterprises whose pollution fee standard was not adjusted during the sample period. Considering that some characteristics between enterprises are significantly different and will affect the correctness of the results, suitable samples are further selected for comparison [40]. We adopted the propensity score matching DID (PSM-DID) method as one of the robustness tests.

**Figure 5:**
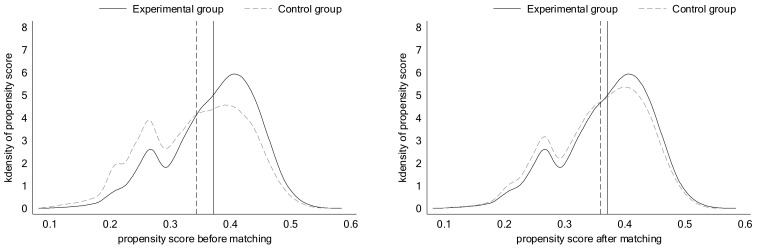
Before matching and after matching.

Specifically, referring to the method of Wu and Wang [41], the method of year-by-year matching was adopted, and caliper nearest neighbor matching was used for 1:4 matching, that is, a treatment group matches at most four control groups. A caliper of 0.001 means that a propensity score match between ±0.001 is selected. Among them, considering that the selection of covariates should be related to both the explained and core explanatory variables, the covariates of this paper were selected from the NH3−N emissions, industrial wastewater emission volume, gross industrial output value, industrial water consumption, and industrial wastewater treatment volume. The matching results are shown in Figure 5.

Figure 5 shows that before matching, the mean difference between the propensity scores of the experimental and control groups is relatively large. However, after matching, the gap is significantly smaller, indicating that the matching effect is better.

Column (a) of Table 4 shows the regression results of the PSM-DID method. The results are basically consistent with those of the benchmark regression. This further proves the reliability of the conclusion in this paper. Referring to the practice of Xu and Jang [42], a more stringent caliper (caliper of 0.0001) was used for testing, and the K neighbors were matched 1 to 2 (i.e., the ratio of the number of experimental groups to the number of control groups). The results are shown in column (b) of Table 4. The results also show the consistency with the benchmark regression.

#### 4.2.2. Placebo Test

**Figure 6:**
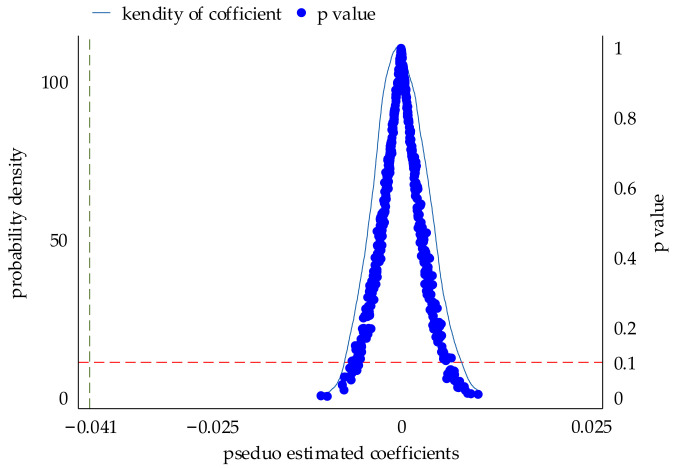
Results of the Placebo test.

In fact, in addition to the impact of the pollution fee reform on COD emissions, there may be other factors that affect COD emissions and make the results inaccurate. Therefore, this paper randomized the treatment group samples and policy implementation time and then conducted a placebo test. The specific operation refers to the practice of Wang et al. [43]: first, randomly generate the sample order, and then shuffle the original policy shock dummy variable according to this order, thus generating a new policy shock dummy variable. Then make this new policy shock dummy variable randomly match all enterprises in the original sample, so that the successful matching is used as the experimental group, and the unsuccessful ones are used as the control group. Regression sampling was performed 500 times without release, and the regression coefficients, standard errors, and p values were saved and plotted. The placebo test result can be seen in Figure 6.

As seen in Figure 6, the solid line represents the kernel density distribution of the coefficients, and the blue circles represent the p value of the coefficients. Most of the virtual regression coefficients are approximately 0, and the real estimated value (i.e., −0.041) is an obvious outlier. Most of the *p* values are above 10%, which is not significant and shows that the real estimation results are not obtained by chance. Therefore, the influence of other policies can be largely excluded.

#### 4.2.3. Excluding the Data of Municipalities

Municipalities are the most important provincial administrative bodies in many countries [44]. The municipalities in China, due to the particularity of politics, economy, and geographical location, place more pressure on urban water security (such as water pollution and depletion) than other cities [45]. This may bias the results of this study, so this paper removed the sample data from four municipalities (i.e., Beijing, Tianjin, Shanghai and Chongqing) and re-performed the regression analysis. The regression results are shown in column (a) of Table 5.

After excluding the data of the four municipalities, the policy still has negative effects on the COD emissions of enterprises and is established at a 10% significance level. This is consistent with the benchmark regression.

#### 4.2.4. Excluding the Impact of Low-Carbon City Pilot Policies

To protect the ecological environment, the Chinese government issued a list of low-carbon city pilot policies in 2010, 2012, and 2017. This pilot policy helps to achieve industrial structure upgrading and has significantly improved green growth [46]. Therefore, the pilot policy may bias the results of this study. Thus, cities that are covered by the policy during the sample period of this paper were removed, and then multiperiod DID regression was performed. The regression results are shown in column (b) of Table 5.

After excluding the sample of the enterprises that have implemented low-carbon city pilot policies, the reform of water pollution fees has negative effects on COD emissions and is established at a 1% significance level. This conclusion is also consistent with the benchmark regression.

### 4.3. Heterogeneity Analysis

#### 4.3.1. Divided into Resource-Based and Nonresource-Based Cities

Resource-based cities have injected a strong impetus into their economic development, but the overexploitation of resources in resource-based cities leads to serious water pollution problems and brings great pressure to the environmental governance of governments [47]. Achieving sustainable environmental and economic development in resource-based cities has attracted the attention of governments [48]. This study explored the influences of the pollution fee reform on water pollution in resource- and nonresource-based cities. With reference to the division of resource-based cities in the National Sustainable Development Plan for Resource-based Cities (2013–2020), the study samples were divided into resource- and nonresource-based cities. The results can be seen in Table 6. Pollution fee reform can significantly reduce COD emissions in resource-based cities. However, the effect is not significant for nonresource-based cities. For resource-based cities, the coefficient is −0.082, which means that compared with enterprises with no change in pollution fee standards, enterprises in the cities where the standard is changed reduce COD emissions by an average of 8.2%. The reason may be that resource-based cities have high pollutant emissions and strict environmental supervision, which leads to the obvious emission reduction effect of the pollution fee reform.

#### 4.3.2. Location of Enterprises

In terms of temperature, there is a significant difference between northern and southern China in the winter. Furthermore, the northern part of China often uses winter heating, which is prone to pollution [49]. In terms of rainfall, generally speaking, it is believed that there is more rainfall in southern China than in northern China [50]. Moreover, the industrial development of northern and southern China is different. The northern region is highly dependent on investment-driven heavy industry, and its economic development lacks resilience [51]. Therefore, referring to the practice of Xu [52], the “Qinling–Huaihe” line was taken as the dividing line between northern and southern China. We explored the different impacts of pollution fee reform in northern and southern China. The results are shown in Table 7.

The reform of water pollution fees encourages enterprises in northern China to engage in emission reduction behaviors, while enterprises in southern China are not sensitive to the reform of water pollution fees. For the sample of northern China, the coefficient is −0.14, which means that compared with enterprises with no change in pollution fee standards, enterprises in the region where the standard is changed reduce COD emissions by an average of 14%. The reason may be that northern China is highly dependent on heavy-industry enterprises to develop its economy, and its pollution emissions are relatively serious, so it is more sensitive to the reform of water pollution fees.

### 4.4. Mechanism Analysis

According to the above theoretical analysis, when enterprises face environmental regulation, the ways to reduce emissions include production reduction, pollutant process control, or end-of-pipe control. To explore the main emission reduction methods adopted by enterprises, this paper conducted a regression analysis of the three methods. Among them, the process control is represented as the ratio of the COD generation to the gross industrial output value of the enterprise. The smaller the index, the better the process control. End-of-pipe control is expressed by the ratio of COD removal to COD generation. The larger the index, the better the end-of-pipe control. Due to the lack of data on COD removal, this paper used the difference value between COD generation and COD emissions to indirectly represent COD removal. In the regression of the enterprise output value, the generation amounts of COD and NH3−N were added because the combined generation amount of these two pollutants more directly reflects the output value of the enterprise than the emissions, and the first-order lag term of the gross industrial output value was added at the same time. The regression results can be seen in Table 8.

It can be found that the standard increase in COD pollution fees has a significant impact on end-of-pipe control and the total industrial output value and has no significant impact on pollutant process control. This shows that enterprises tend to choose end-of-pipe control and reduce production when facing the reform of water pollution fee standards. Hypothesis 2 was verified. The main reason is that the process control of pollutants, such as production process improvement and resource recycling, has a long cycle, large investment, and relatively high risk. Therefore, enterprises are more inclined to choose end-of-pipe control (such as the introduction of pollution treatment equipment, etc.) or directly reduce production. However, from a long-term perspective, in addition to reducing pollutant emissions, process control can also save resources and improve resource utilization efficiency, which is conducive to the improvement of corporate competitiveness. At the same time, it is necessary to properly combine pollutant process control and end-of-pipe control, and enterprises cannot attend to one thing and lose sight of another if they seek to play a greater role [53].

## 5. Discussion

This paper found that the improvement of pollution fee collection standards can promote the COD reduction of enterprises. The conclusion is consistent with Dong et al.’s, who believe that pollution charges have a suppressive effect on COD emissions [26]. However, Dong et al. used provincial panel data and used the total amount of pollution fees as an explanatory variable, whereas this paper used enterprise-level data and pollution fee reform to explore the relationship between pollution fees and COD emissions. In terms of research methods, this paper drew on the practice of Liu et al. and adopted a multiperiod DID method [40]. The difference is that Liu et al. studied the impact of low-carbon city pilot policies on CO_2_ emissions, whereas this paper analyzed the impact of pollution fee reform on COD emissions, and the multiperiod DID can capture the policy effects brought about by inconsistent policy occurrence.

Drawing on Ren et al.’s practice, the impact of pollution fee reform on pollutant emissions is divided into three channels, namely process control, end-of-pipe control, and output value [39]. This paper found that enterprises choose to reduce production and end-of-pipe control when facing pollution fee reform, which is slightly different from Ren et al.’s research. Ren et al. found that enterprises choose to reduce production when facing pollution fee reform. The reason for the difference may be that Ren’s research subject is air pollution. In the early stage of the pollution fee reform, the government focused on air pollution, so the levy standard for water pollution was lower than that for air pollution. Therefore, enterprises would be more inclined to choose direct production reduction when facing air pollution charges.

In addition, this paper also found that after dividing the samples into southern and northern China, the impact of pollution fee reform on water-pollutant emission in northern China was more significant than that in southern China. The reason may be that heavy industries were mostly located in northern China [49], and the pollution level was higher than that in southern China, which attracted more attention from local governments. Therefore, it is more sensitive to the reform of water pollution fees in northern China.

The main limitations of the study are as follows: (1) in terms of methodology, multiperiod DID used in this paper cannot capture the degree of change in the reform of pollution fees. In some provinces, the collection standard of water-pollutant emission increased by a large margin, which may lead to different reform effects. (2) this paper used micro-enterprise-level data to analyze the impact of pollution fee reform, which cannot portray the macro level (e.g., the city level). It is also significant to analyze the difference of the impact of pollution fee reform among different cities, which is also the research field we will pay attention to in the future.

## 6. Conclusions and Policy Implications

This paper took the pollution fee reform in China as a quasi-natural experiment, selected sample data of China’s manufacturing enterprises from 2006 to 2013, and systematically studied the impact of pollution fee reform on enterprises’ emissions. Raising the standard of COD pollution fees is conducive to the emission reduction of enterprises. There are differences in the impact of water pollution fee reform in different regions. Specifically, raising the standard of water pollution fees is significantly beneficial to the emission reduction of enterprises in northern China and resource-based cities. However, the effect is not significant in southern China and nonresource-based cities. Further analysis of its mechanism of behavior shows that enterprises are more inclined to choose process control or directly reduce production rather than the process control of pollutants.

Based on the above conclusions, this paper put forward the following policy suggestions. First, although the pollution fee reform has generally promoted the emission reduction of manufacturing enterprises, the incentive effect for enterprises is insufficient in some regions. The main reason is that the standard of pollution fees is still low, and some local governments lack supervision, so they cannot effectively force enterprises to reduce emissions. Following the “polluter pays principle”, the government should gradually increase the standard of the environmental protection tax, promoting the internalization of all external costs of emissions by enterprises, and rely on the authority of taxation to force enterprises to reduce emissions. Second, to achieve environmental and economic sustainable development, and improve the competitiveness of enterprises, pollution process control is necessary. The government should increase the support for enterprises, such as implementing preferential tax policies, to encourage enterprises to carry out green process transformation and recycling of resources, which improve enterprise productivity while reducing emissions.

## Figures and Tables

**Table 1:** Descriptive statistics.

Variable	Definition	Units	Obs.	Mean	Std. Dev.	Min.	Max.
LnCOD	COD emissions	kilograms	326,368	8.344	2.249	2.303	13.726
COD_DID	Policy dummy variable	-	326,368	0.227	0.419	0	1
LnCOD_g	COD generation	kilograms	326,368	9.422	2.571	2.674	15.428
LnGiov	Gross industrial output value	10 thousand Chinese yuan	326,368	8.142	1.879	3.447	13.171
LnIwc	Industrial water consumption	tons	326,368	11.184	2.187	5.635	17.487
LnIwev	Industrial wastewater emissions volume	tons	326,368	10.510	2.047	4.913	15.286
LnIwtv	Industrial wastewater treatment volume	tons	326,368	7.790	5.229	0	15.594
LnNH3-N_e	${\mathrm{NH}}_{3}$−N emissions	kilograms	326,368	4.035	3.276	0	11.043
LnNH3-N_g	${\mathrm{NH}}_{3}$−N generation	kilograms	326,368	4.542	3.621	0	12.121

**Table 2:** Adjustment time of COD pollution fee collection standards during the sample period.

Province Name	Time	Province Name	Time
Jiangsu Province	July 2007	Yunnan Province	January 2009
Shanghai Province	June 2008	Guangdong Province	April 2010
Hebei Province	July 2008	Liaoning Province	August 2010
Shandong Province	July 2008	Xinjiang Province	August 2012

**Table 3:** Benchmark regression.

Variable	LnCOD	LnCOD
	(a)	(b)
COD_DID	−0.053 *	−0.041 **
	(−1.791)	(−2.032)
LnGiov		0.017 ***
		(4.080)
LnIwc		0.035 ***
		(4.878)
LnIwtv		−0.024 ***
		(−9.131)
LnIwe		0.849 ***
		(79.333)
LnNH3-N_e		0.056 ***
		(9.647)
_cons	8.357 ***	−1.146 ***
	(1225.876)	(−12.699)
Individual fixed effect	Yes	Yes
Time fixed effect	Yes	Yes
Industrial-Time fixed effect	Yes	Yes
Observations	319,895	305,872
R-squared	0.770	0.887

Note: *t* statistics in parentheses. * *p* < 0.10, ** *p* < 0.05, *** *p* < 0.01.

**Table 4:** Regression results of PSM-DID method.

Variable	LnCOD (0.001; 1:4)	LnCOD (0.0001; 1:2)
	(a)	(b)
COD_DID	−0.041 **	−0.038 *
	(−2.028)	(−1.904)
LnGiov	0.017 ***	0.017 ***
	(4.068)	(3.916)
LnIwc	0.034 ***	0.030 ***
	(4.958)	(4.429)
LnIwtv	−0.024 ***	−0.024 ***
	(−9.104)	(−8.963)
LnIwe	0.850 ***	0.855 ***
	(81.163)	(81.846)
LnNH3-N_e	0.056 ***	0.056 ***
_cons	(9.657) −1.146 *** (−12.676)	(9.768) −1.153 *** (−12.596)
Individual fixed effect Time fixed effect Industrial-Time fixed effect	Yes Yes Yes	Yes Yes Yes
Observations	305,620	300,801
R-squared	0.887	0.885

Note: *t* statistics in parentheses. * *p* < 0.10, ** *p* < 0.05, *** *p* < 0.01.

**Table 5:** Excluding four municipalities and excluding the impact of low-carbon policies.

	LnCOD	LnCOD
	(a)	(b)
COD_DID	−0.038 *	−0.120 ***
	(−1.721)	(−7.544)
LnGiov	0.017 ***	0.017 ***
	(3.976)	(3.877)
LnIwc	0.034 ***	0.037 ***
	(4.361)	(4.269)
LnIwtv	−0.024 ***	−0.025 ***
	(−8.703)	(−8.616)
LnIwe	0.852 ***	0.852 ***
	(77.343)	(69.275)
LnNH3-N_e	0.054 ***	0.049 ***
	(9.363)	(8.547)
_cons	−1.133 ***	−1.117 ***
	(−12.087)	(−11.902)
Individual_fixed_effect	Yes	Yes
Time_fixed_effect	Yes	Yes
Industrial_Time_fixed_effect	Yes	Yes
Observations	282,624	226,361
R-squared	0.889	0.894

Note: *t* statistics in parentheses. * *p* < 0.10, *** *p* < 0.01.

**Table 6:** Impact of pollution fee reform inside or outside resource-based cities.

	Resource-Based Cities	Non-Resource-Based Cities
	(a)	(b)
COD_DID	−0.082 ***	−0.026
	(−3.072)	(−1.155)
LnGiov	0.024 ***	0.015 ***
	(2.787)	(3.337)
LnIwc	0.056 ***	0.026 ***
	(3.709)	(3.436)
LnIwtv	−0.025 ***	−0.024 ***
	(−7.558)	(−8.162)
LnIwe	0.773 ***	0.876 ***
	(41.458)	(80.859)
LnNH3-N_e	0.053 ***	0.057 ***
	(7.829)	(9.193)
_cons	−0.406 ***	−1.368 ***
	(−2.773)	(−16.333)
Individual_fixed_effect	Yes	Yes
Time_fixed_effect	Yes	Yes
Industrial_Time_fixed_effect	Yes	Yes
N	59,536	245,554
R-squared	0.868	0.894

Note: *t* statistics in parentheses. *** *p* < 0.01.

**Table 7:** Location heterogeneities of the impact of pollution fee reform.

Variable	Southern China	Northern China
	(a)	(b)
COD_DID	0.016	−0.140 ***
	(0.565)	(−6.230)
LnGiov	0.021 ***	0.007
	(4.098)	(0.911)
LnIwc	0.034 ***	0.041 ***
	(4.336)	(3.171)
LnIwtv	−0.024 ***	−0.025 ***
	(−7.562)	(−8.617)
LnIwe	0.859 ***	0.822 ***
	(74.766)	(48.709)
LnNH3-N_e	0.052 ***	0.067 ***
_cons	(7.940) −1.329 *** (−14.947)	(11.203) −0.683 *** (−5.281)
Individual fixed effects Time fixed effect Industrial-Time fixed effect	Yes Yes Yes	Yes Yes Yes
Observations	225,881	79,497
R-squared	0.892	0.878

Note: *t* statistics in parentheses. *** *p* < 0.01.

**Table 8:** Mechanism analysis.

Variable	Process Control	End-of-Pipe Control	Output Value
	(a)	(b)	(c)
COD_DID	0.018	0.011 **	−0.024 **
	(0.698)	(2.003)	(−2.263)
LnIwc	−0.029 ***	0.009 ***	0.113 ***
	(−3.231)	(4.932)	(9.994)
LnIwtv	0.031 ***	0.028 ***	0.001
	(16.428)	(25.451)	(0.523)
LnIwe	0.646 ***	−0.037 ***	0.024 ***
	(34.865)	(−17.187)	(5.375)
LnNH3-N_e	0.040 ***	−0.002 ***	
	(9.207)	(−4.355)	
LnGiov		0.007 ***	
		(5.417)	
LnCOD_g			0.021 ***
			(6.330)
LnNH3-N_g			0.003 ***
			(2.666)
L.LnGiov			0.205 ***
_cons	−5.571 *** (−28.355)	0.462 *** (31.592)	(35.657) 4.800 *** (30.957)
Individual fixed effect Time fixed effect Industrial-Time fixed effect	Yes Yes Yes	Yes Yes Yes	Yes Yes Yes
Observations	313,560	308,262	233,714
R-squared	0.829	0.635	0.902

Note: *t* statistics in parentheses. ** *p* < 0.05, *** *p* < 0.01.

## Data Availability

Not applicable.
